# The Role of Hypoxia in Orthodontic Tooth Movement

**DOI:** 10.1155/2013/841840

**Published:** 2013-10-21

**Authors:** A. Niklas, P. Proff, M. Gosau, P. Römer

**Affiliations:** Department of Orthodontics, University Medical Center Regensburg, 93053 Regensburg, Germany

## Abstract

Orthodontic forces are known to have various effects on the alveolar process, such as cell deformation, inflammation, and circulatory disturbances. Each of these conditions affecting cell differentiation, cell repair, and cell migration, is driven by numerous molecular and inflammatory mediators. As a result, bone remodeling is induced, facilitating orthodontic tooth movement. However, orthodontic forces not only have cellular effects but also induce vascular changes. Orthodontic forces are known to occlude periodontal ligament vessels on the pressure side of the dental root, decreasing the blood perfusion of the tissue. This condition is accompanied by hypoxia, which is known to either affect cell proliferation or induce apoptosis, depending on the oxygen gradient. Because upregulated tissue proliferation rates are often accompanied by angiogenesis, hypoxia may be assumed to fundamentally contribute to bone remodeling processes during orthodontic treatment.

## 1. Introduction

The connective tissue between teeth and bone termed periodontal ligament (PDL) is a highly differentiated ligamentous apparatus designed to convert chewing forces into tensile forces to transmit them to the alveolar bone. The dental root is entirely coated with cementum, which consists of cementocytes, mineralized hard tissue, and collagen fibers. Two types of collagen fibers can be found in the cementum: intrinsic fibers (Ebner fibrils) that run around the root in a circle and extrinsic fibers (Sharpey's fibers) that are inserted into the cementum and run to the inner walls of the tooth socket [1]. In the rest position, these fibers are corrugated and allow tooth mobility up to a certain extent; during physiological mechanical load, however, these fibers straighten. The collagen fiber bundles of the PDL are surrounded by an incompressible matrix composed of glycosaminoglycan, glycoproteins, and glycolipids. The periodontal ligament mainly consists of fibroblasts and fibrocytes but also of osteoblasts, osteoclasts, macrophages, cementoblasts, and progenitor cells [2, 3]. 

Loose connective tissue is found in the apical region of the teeth and in the small gaps between the collagen fiber bundles of the PDL. The connective tissue is hosting nerves as well as blood and lymphatic vessels. Several mechanisms are assumed to have a cushioning effect on ensuring the nutrition of the tooth-PDL-system during physiological mechanical loading. Shock-absorbing effects are attributed to the resilience of the dental hard tissue and the periodontal space filled with tissue fluid [4, 5]. In the case of minor external impact, damage to the periodontium is prevented by the elongation of collagen fibers of up to 5% of their initial length and the elastic deformation of tooth sockets. These mechanisms are also thought to be responsible for the fact that light-intermitting orthodontic forces do not induce cellular effects [4]. Fibroblasts of the periodontal ligament are known to build new collagen fibrils and differentiate into cementoblasts, osteoblasts, or osteoclasts. Because of its rich cellular population and good renewal capability, the PDL can quickly adapt to moderate external influences, such as orthodontic forces [3]. 

This fact considerably contributes to the remodeling capabilities of the alveolar bone. The alveolar process of the maxilla consists of a thin cortical bone plate covered with a thin tough membrane called the periosteum. Tooth sockets are formed by a perforated bone layer termed lamina cribriformis. The space between the cortical bone and the tooth socket is filled with cancellous bone that mainly consists of cross-linked bone trabeculae with a rather inhomogeneous structure. In contrast, mandibular bones are very dense cortical bones [1]. Bone tissue is fundamentally based on collagen and hardened by the deposition of calcium apatite [6]. Osteoblasts, which are responsible for bone formation and calcification, are in balance with bone-resorbing osteoclasts [7]. The functional interaction of these three cell types is essential for bone remodeling during orthodontic tooth movement, and their activity is strictly controlled by molecular mediators [8–11]. Although our knowledge on the molecular and cellular regulation of bone formation [8–10, 12–16] has increased in recent years, many questions remain with regard to the mechanisms facilitating orthodontic tooth movement. 

## 2. Stages of Orthodontic Tooth Movement

Malocclusions are treated by attaching orthodontic appliances (loose or fixed) to achieve eugnathia. The process of orthodontic tooth movement with continuous forces is divided into three stages, namely, the initial phase, the lag phase, and the postlag phase. During the first days (initial phase), rapid tooth movement can be observed caused by the cushioning effects of the periodontal system described above [5, 17]. For example, the periodontal gap in dogs is distinctly compressed after 3 to 6 days of force application with forces up to 30 N [4]. In the case of prolonged and enhanced orthodontic force application, tooth movement often stops for up to 20 days, a condition termed lag phase [17]. During this stage, the periodontal tissue and alveolar bone on the pressure side of the dental root are suffering from circulatory disturbances [14]. Affected parts of the PDL and the adjacent alveolar bone show partial necrosis; histologically, they appear similar to hyalinized tissue [18]. During the lag phase, resorption of tooth sockets only takes place in terms of undermining resorption of the lamina cribriformis, starting in the vital bone. If the undermining resorption is exceeded at a certain stage, the alveolus will collapse, resulting in sudden tooth movement. In contrast, the postlag phase or late phase is coined by direct resorption of the socket, allowing continuous tooth movement [14, 17]. An intact periodontal circulatory system prevents alveolar tissues from hyalinization. Therefore, orthodontists will always try to achieve a smooth transition from the initial phase to the postlag phase by skipping the lag phase [4]. 

## 3. Molecular and Cellular Regulation of Tooth Movement

By compressing the PDL, orthodontic forces induce cell deformation in alveolar tissues, stimulating mechanosensitive ion channels and receptors in the cell membrane [2]. Periodontal cells seem to react to mechanical stimulation by upregulating cellular mediators, such as cyclic AMP (cAMP), that induce proteinkinases to catalyze the phosphorylation of mediator proteins [19]. After several steps, the information about the mechanical stimulation reaches the nucleus, in which two main pathways can be triggered. DNA replication leading to cell proliferation can be initiated, and cell differentiation can be induced. The activity of genes related to bone growth and cell reprogramming is modulated by the phosphorylation of the transcription factors, such as c-Jun und c-Fos. Important cellular mediators, such as protein kinase C (PKC), and inflammation mediators, such as prostaglandin E_2 _ (PGE_2_), are generated and released into the cytoplasm [2]. In the case of inflammation, cyclooxygenase 2 (COX-2) is stimulated to produce prostaglandin H_2 _ (PGH_2_), which is followed by the conversion of PGH_2_ to PGE_2_ by prostaglandin E synthase. By binding to the two main PGE_2_ receptors E2 and E4, PGE_2_ has been reported to activate adenylate cyclase in human PDL cells and thus seems to stimulate bone resorption [20]. 

However, the upregulation of COX-2 and the expression of PGE_2_ are known to play an important role in bone resorption [8, 21]. De Carlos et al. [22] found significantly inhibited tooth movement as a result of COX-2 inhibitor application in rats [22]. The literature reports that the inhibition of prostaglandin E2 by vitamin K2 may also inhibit osteoclastogenesis [23]. Inflammatory processes stimulate mononuclear phagocytic cells, such as macrophages, that secret proinflammatory cytokines, for example, interleukin-1*β* (IL-1*β*) and interleukin-6 (IL-6). Interleukins are part of the peptide hormone family and internal body messenger elements for immune cells. 

Nevertheless, these proinflammatory cytokines are also known to enhance the secretion of prostaglandins [12, 16]. During orthodontic force application, these cytokines are assumed to regulate the secretion of the receptor activator of the nuclear factor kappa ligand (RANKL) by osteoblasts and PDL cells in periodontal tissues [16, 24]. RANKL is a ligand to the receptor activator of the nuclear factor kappa (RANK) that is localized on the surface of osteoclast precursor cells as well as on mature osteoclasts [25, 26]. If RANKL binds to RANK, osteoclast precursor cells fuse and differentiate into active multinucleated osteoclasts responsible for bone resorption [11, 16, 27]. As long as RANKL is expressed and the equilibrium of RANKL and osteoprotegerin (OPG) is pushed to the side of RANKL, RANK stays activated and osteoclasts proceed with bone resorption. To stop resorption, osteoblasts increase OPG production, which is a competitive receptor for RANKL and related to the tumor necrosis factor receptor (TNFR) [16]. In addition to the inhibition of RANKL, OPG has also the ability to block mature osteoclasts or to downregulate osteoclastogenesis [25]. Nakao et al. [24] showed that OPG mRNA is downregulated and force-dependent as well as time-dependent, when human PDL cell cultures are charged with compressive load. In contrast, the expression of RANKL mRNA was induced in compressed cell populations and inconspicuous in unstimulated PDL cells [24]. Thus, the occurrence of osteoclastogenesis and bone resorption seems to depend on the balance of RANKL and OPG [11, 25, 26]. 

## 4. Hypoxia and HIF-1***α*** in Orthodontic Tooth Movement 

Hypoxia describes oxygen deficiency in tissue due to oxygen partial pressure reduced beyond the physiologic level [28]. Physiologic oxygen partial pressure in tissue depends on the localization of the tissue and the age of the patient. In pulmonary veins, for example, the oxygen partial pressure fluctuates between 80 and 100 mmHg, whereas 40 mmHg can be found in terminal capillary networks [29]. Hypoxia influences cellular energy levels by reducing glycolytic activity and ATP production. The cells respond to hypoxia by expressing cellular mediators, particularly the hypoxia-inducible factor 1 (HIF-1), a heterodimer composed of HIF-1*α* and HIF-1*β*. The formation of HIF-1 is limited by the subunit HIF-1*α*. Although HIF-1*α* is expressed during both normoxia and hypoxia, this subunit is unstable in normoxia. Hydroxylated by HIF hydroxylases under normoxic conditions, the proteolytic degradation of HIF-1*α* is facilitated by the binding of the Von Hippel-Lindau tumor suppressor protein (pVHL). This protein induces the attachment of a polyubiquitin chain to HIF-1*α*, thus permitting the anchorage of HIF-1*α* to the proteasome and finally leading to the degradation of HIF-1*α*. During hypoxia, the stabilized HIF-1*α* aggregates and binds to HIF-1*β*, creating the active transcription factor HIF-1 that can promote angiogenesis, stimulate cell proliferation, and is able to prevent cell death. But HIF-1 also induces apoptosis to inhibit hypoxia-induced mutations in cells [28, 30, 31].

Matrix metalloproteinases (MMPs) are known to be important for angiogenesis because of their contribution to the degradation of the vascular basal membrane, leading to endothelial cell migration [32]. During tooth movement, periostin is mainly expressed on the pressure side of the PDL [33] and is upregulated by hypoxia [15]. 

Watanabe et al. [15] treated PDL cells with desferrioxamin—used for hypoxia simulation in cell culture—for 24 h. The investigators could show that HIF-1*α* was induced and subsequently found the upregulation of periostin but could not clarify if this upregulation was induced directly or indirectly by HIF-1*α*. Particularly noteworthy was that periostin seems to contribute to the secretion and transcription regulation of MMP-2 [15]. 

By coculturing human primary peripheral blood mononuclear cells (PBMNCs) with and without osteoblasts (OBs) under normoxic (21.0%) and hypoxic (2.5% and 5.0%) conditions, Dandajena et al. [34] showed that PBMNC can differentiate into functional osteoclasts (OCs) when triggered by hypoxia. After the induction of a 2.5-fold increase of HIF-1*α*, the authors found a steep slope of the vascular endothelial growth factor (VEGF) between 24 h and 72 h of exposure, and the upregulation of RANKL directly correlated with the HIF-1*α* level [34].

VEGF is known as one of the most important mitogen that induces angiogenesis. By adhering to receptors of endothelial cells, VEGF activates signal cascades, resulting in a broad variety of cellular and vascular reactions [35]. Via the expression of nitric oxide (NO), VEGF is also able to indirectly modulate vasodilation. NO is not only a fundamental biological messenger but also a radical gas. As a signaling molecule, NO plays a crucial role in a variety of biological processes, for instance, the regulation of vasodilation, blood flow, and inflammation [36]. Thus, orthodontic tooth movement not only has cellular effects, such as the above-mentioned activation of osteoclasts or osteoblasts, but also induces vascular changes [9, 37]. 

Khouw and Goldhaber [37] investigated vascular changes in the PDL after 1, 3, and 7 days by applying tipping forces onto the teeth of rhesus monkeys and German shepherd dogs. The authors observed similar changes in both species for all test conditions and therefore did not discuss the results for each species separately. After 24 h of force application, vessels on the tension side of the root were widened, whereas blood vessels on the pressure side, particularly above the rotation center of the root, showed partial or complete occlusion. Results for the tension side in the group of 72 h force application appeared similar to those after 24 h, but osteoblasts were accompanied by new bone formation. Vascularisation of the PDL was still suppressed in the areas of pressure, and no active bone resorption could be observed. After seven days of force application, the authors found new bone formation throughout all tension areas. Although all blood vessels were dilated, no angiogenesis could be observed at the tension site. The alveolar bone showed surface resorption as well as undermining resorption in the areas of pressure, and increased numbers of new blood vessels were found near the areas affected by resorption [37].

**Figure 1:**
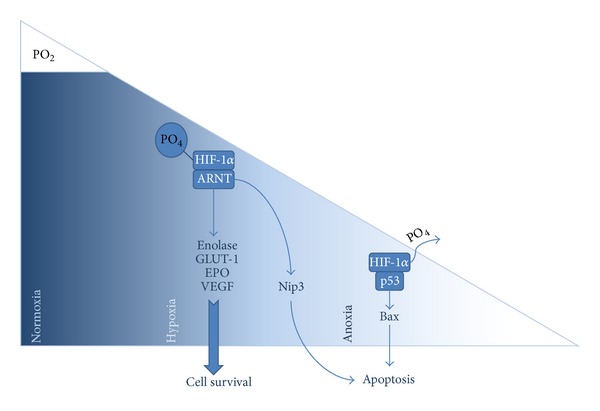
Sketch of hypoxia induced pathways mediated by HIF-1*α*, leading to either cell survival or apoptosis [31].

These findings correspond to the fact that the occlusion of blood vessels naturally decreases blood supply, resulting in hypoxia in areas of pressure [9, 37]. Subsequently, hypoxia induces the formation of the active transcription factor HIF-1 and activates genes encoding VEGF [38]. In cells with chronic or extreme hypoxia, however, the protective effect of HIF-1*α* ceases, resulting in cell apoptosis. Interestingly, two pathways have been discovered by which HIF-1*α* itself seems to induce apoptosis. On the one hand, hypoxia triggers the expression of the proapoptotic nineteen kD interacting protein-3 (Nip3), which seems to be strongly related to the presence of HIF-1*α*. On the other hand, hypoxia stabilizes the p53 tumor suppressor protein. This transcription factor is able to activate genes, such as the apoptosis-regulator Bax, that initiate cell death or stop proliferation (Figure 1).

According to Nip3, p53 also relies on the presence of HIF-1*α*, since none of the two transcription factors can be found in cells without HIF-1*α* [31]. PDL cells suffering hypoxia release biochemical mediators, such as the CC chemokine ligand 2 (CCL2). This mediator can be particularly found in the resorption site after the application of orthodontic forces. CCL2 activates the CC chemokine receptor 2 (CCR2) that is mainly localized on leucocytes, monocytes or macrophages, and lymphocytes. Chemokines are assumed to induce leucocyte chemotaxis and cellular activation, leading to cell proliferation and angiogenesis. But CCL2 is also known to trigger protective effects against cell death, particularly in the case of mild hypoxia [9].

Kitase et al. [9] investigated the gene expression in PDL cells and exposed primary PDL cells to hypoxia by reducing the total oxygen concentration to less than 1% for 96 h. The investigators also studied the protective effect of recombinant human CCL2. After incubating human PDL cells for 12 h with less than 1% oxygen concentration, Kitase et al. [9] identified 11 hypoxia-responsive genes. Particularly the insulin-like growth factor-binding protein 3 (IGFBP3) and CCR2 were upregulated under hypoxic conditions, whereas CCL2 was drastically downregulated. The authors were able to prove that the negative effects of hypoxia in PDL cells can be prevented by adding external CCL2 [9]. They also reported a 55.4% increase in nonviable cells due to hypoxia, whereas the addition of 10 ng/mL of CCL2 significantly decreased nonviable cells to 9.8%. Kitase et al. also proposed that IGFBP3 is one of the main mediators responsible for cell death under hypoxia [9].

Tuncay et al. [39] reported that light hypoxic conditions (10% O_2_) seemed to increase the proliferation rate of osteoblast-enriched cultures *in vitro*. In contrast, hyperoxia (90% O_2_) even showed a reversed effect, because changing oxygen tension from low to high immediately results in a drastic decrease in the proliferation rate [39]. In summary, hypoxia seems to fundamentally contribute to bone remodeling processes [9, 34, 39].

## 5. Influence of Orthodontic Forces on Tooth Movement and Root Resorption

Resorption of permanent teeth is always regarded as a pathologic process, and the etiology of root resorption is not yet completely understood. In general, root resorption can be located in three different areas of the dental root and is classified as lateral resorption, apical resorption, and internal resorption. Teeth suffering trauma may show all three types of root resorption. After replantation, lateral root resorption can be seen in avulsed teeth, particularly after damage to the periodontal tissue. Apical or internal resorption is usually a side effect of pulpal inflammation. Root resorption can also occur in the case of neoplasia, such as pulp polyps or tumors. A special case is the undermining resorption of permanent teeth, which can occur in impacted teeth or teeth with abnormal eruption paths [40]. Depending on the individual predisposition, idiopathic resorption may even occur without any prior trauma [40, 41]. 

But root resorption—apical as well as lateral—is also an undesirable risk of orthodontic treatment [40–43]. Some orthodontic treatment factors are especially in discussion for being related to root resorption, such as force level, tipping forces, intrusion or extrusion of teeth. But also anatomical factors such as tooth root morphology might be relevant [44, 45]. By applying a continuous force of 50 cN, respectively, 200 cN for the duration of seven weeks on human premolars, Owman-Moll et al. [46] observed a mean tooth displacement of 3.5 mm for 50 cN and 5.1 mm for the group with four times higher force levels. However, no significant difference could be found comparing both force levels regarding the occurrence and severity of root resorption. Instead they found all test teeth suffering root resorption independently from the applied force but with large differences between individuals [46]. In order to investigate whether intermitting or continuous forces are harmful to the dental root, another group applied tipping forces of 100 g on human premolars for 12 and 24 hours a day. After nine weeks, the teeth were extracted and prepared for scanning electron microscopy (SEM). The authors could show that continuous force application resulted in a higher percentage of root resorption, whether in the palatal, buccal, or apical region. Even though they also found distinct individual differences in the severity of root resorption, the authors verified root resorption on all test teeth [47]. As intrusion of teeth is considered as a high risk treatment, often resulting in root resorptions, Harris et al. [48] conducted a study in which 54 human premolars were intruded for 28 days using either low (25 g) or heavy (225 g) forces. The analysation of X-ray microtomography images of the extracted test teeth unveiled that the resorption crater dimension increased according to the increase of the intrusive force [48]. In contrast to that, another group directly compared the effect of intrusive and extrusive forces on the dental root. Using a continuous force of 100 cN on 18 maxillary first premolars in nine patients, they randomly intruded one first premolar and extruded the contralateral premolar in each patient. Subsequently they investigated the root surface of every specimen via SEM images and could show that there was a significant difference between extruded and intruded teeth. While the extruded specimens did not show a significant difference in root resorption compared to the control group, all of the intruded teeth showed obvious signs of resorption in the apical part of the root [49]. It is well known that maxillary incisors with pipette-like or blunt-ended roots seem to be more frequently affected by orthodontically induced root resorption (OIRR) than others, particularly in the apical region [50]. Spurrier et al. [43] found a significant difference between root-filled teeth and vital teeth with regard to their susceptibility for resorption. The authors investigated 43 patients who had received endodontic treatment on one or more anterior teeth prior to orthodontic therapy with a multi-bracket appliance. For each patient, radiographs of the root-filled teeth and the contralateral vital teeth were taken before and after orthodontic therapy. The findings showed a higher number as well as more severe cases of root resorption for vital teeth than for root-filled control teeth. Therefore, the authors suggested that, in one way or the other, the dental pulp might contribute to apical root resorption [43].

## 6. Pulpal Reactions after Orthodontic Tooth Movement

**Figure 2:**
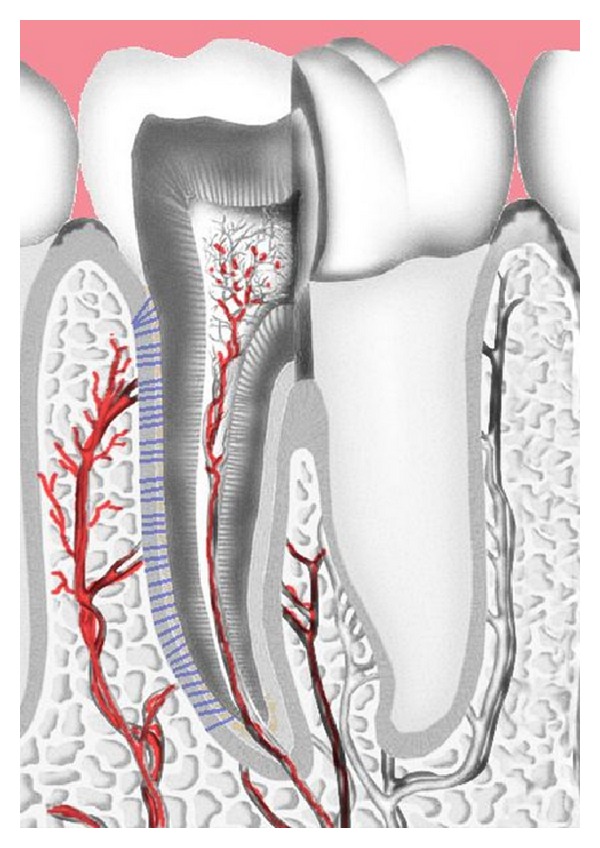
Vascularisation of the dental pulp and the alveolar bone. Blood vessels entering the tooth via the apical constriction.

The dental pulp is divided into two major portions, the crown's pulp and the root. The pulp consists of glycosaminoglycans as a basic element, in which the pulpal cells, such as pulp fibroblasts, odontoblasts, and pulpal stem cells, are embedded [51]. A dense network of arteries, veins, arterioles, venules, and capillaries ensures the high vascularization of the pulp tissue. However, almost all blood vessels enter a tooth via the apical constriction (Figure 2), making it a trouble spot for pulpal blood supply [52, 53]. Pulpal blood vessels are usually accompanied by other functional structures, such as nerves or lymph vessels [54, 55]. The nociceptive innervation of the pulp is mainly based on A-*β*-fibers, A-*δ*-fibers, and C-fibers [56], whereas the vasomotoric nerve fibers of the vegetative nervous system control the muscular tonus of pulpal arterioles and therefore contribute to the regulation of pulpal blood flow [54]. Venules of the dental pulp are known to have very thin walls that tend to collapse in case of high pulpal pressure. In this context, it is also interesting that vasodilation induced by inflammation mediators, such as PGE_2_, seems to have different effects in the dental pulp as in other tissues. By increasing pulpal pressure and therefore hydraulically inducing secondary vasoconstriction, vasodilation might halt the spread of infections, if induced in a very localized area; however, vasodilation may also facilitate necrosis in cases of generalization [54]. Tripuwabhrut et al. [57] induced severe root resorption by applying intermitting tensile loads of 50 g onto 15 first molars in rats for up to 30 days to investigate inflammatory patterns in the dental pulp and the PDL. The investigators found typical signs of inflammation in the compressed PDL, such as immigration of macrophages, monocytes, and dendritic cells. Increased angiogenesis could also be observed in root areas affected by resorption. Nevertheless, no new formation of nerve structures as usually seen in inflamed periodontal and pulpal tissues could be observed [57].

## 7. Conclusion

Orthodontic tooth movement is a process that is based on a variety of mechanisms, such as cell deformation or inflammatory processes. However, some accompanying factors of orthodontic tooth movement, such as force level, tipping forces, intrusion, or extrusion of teeth, but also special anatomical structures might induce circulatory disturbances in the pulpal tissues. Data in the literature shows a possible correlation between orthodontically induced hypoxia in the dental pulp and root resorption. Yet, cellular reactions accompanying orthodontically induced root resorption (OIRR) significantly differ from those seen in inflamed periodontal tissues, indicating that there might be a different pathway of activation. The finding that root-filled teeth seem to be less vulnerable to OIRR than vital teeth requires further investigations into the role of the dental pulp. A full understanding of the mechanism of cellular activation underlying OIRR may facilitate measures to prevent tooth resorption during orthodontic therapy.
